# Description of three new species of *Minucella* (Hemiptera, Cicadellidae, Deltocephalinae, Stegelytrini) from Guizhou Province, China

**DOI:** 10.3897/zookeys.1283.191246

**Published:** 2026-06-24

**Authors:** Ke-Ting Duan, Zi-Zhong Li, Mick D. Webb, Ji-Chun Xing

**Affiliations:** 1 Institute of Entomology, Guizhou University, Guiyang 550025, China Institute of Entomology, Guizhou University Guiyang China; 2 Guizhou Key Laboratory of Agricultural Biosecurity, Guiyang 550025, China Department of Life Sciences, Natural History Museum London United Kingdom; 3 Department of Life Sciences, Natural History Museum, Cromwell Road, London SW7 5BD, UK Guizhou Key Laboratory of Agricultural Biosecurity Guiyang China

**Keywords:** Homoptera, morphology, new taxa, taxonomy

## Abstract

Three new species of the leafhopper genus *Minucella* Wei, Zhang & Webb, 2008 (Cicadellidae, Deltocephalinae, Stegelytrini) are described and illustrated from Guizhou Province, China, *Minucella dilatata***sp. nov**., *M. angusta***sp. nov**., and *M. elongata***sp. nov**. A key to the species of the genus is given together with a revised description and diagnosis. The type specimens of the new species are deposited in the Institute of Entomology, Guizhou University, Guiyang, China (GUGC).

## Introduction

The strikingly marked leafhopper genus *Minucella* Wei, Zhang & Webb, 2008, belongs to the tribe Stegelytrini of the subfamily Deltocephalinae (Hemiptera, Cicadellidae). Its known distribution is currently restricted to China, and since its establishment, only two valid species have been recorded in this genus, namely *M. leucomaculata* (Li & Zhang, 2006) (originally placed in *Placidus*) and *M. divaricata* Wei, Zhang & Webb, 2008. In the present paper, three new species of *Minucella* are described and illustrated from Guizhou Province, China. The type specimens of the new species are deposited in the Institute of Entomology, Guizhou University, Guiyang, China (GUGC). A key is given to separate all species.

## Material and methods

Dry male specimens were used for the descriptions and illustrations. Collected females could not be associated with males (see Generic Remarks). External morphology was observed under a stereoscopic microscope, and characters were measured with an ocular micrometer. The genital segments of the examined specimen were macerated in 10% NaOH. Colour photographs of adult habitus and of the genitalia of specimens were obtained by a Keyence VHX-6000 system. Illustrations were imported into Adobe Photoshop CS8 for labeling and figure composition.

The morphological terminology used in the descriptions mainly follows Li et al. (2011). Absolute measurements, in millimetres (mm), are reported for the body.

## Taxonomy

### Minucella

Taxon classificationAnimaliaHemipteraCicadellidae

Wei, Zhang & Webb, 2008

B7A25C97-2900-5994-B8CC-00008C584463

Minucella Wei, Zhang & Webb, 2008: 33.

#### Type species.

*Minucella divaricata* Wei, Zhang & Webb, 2008.

#### Diagnosis.

**Figure 1.**
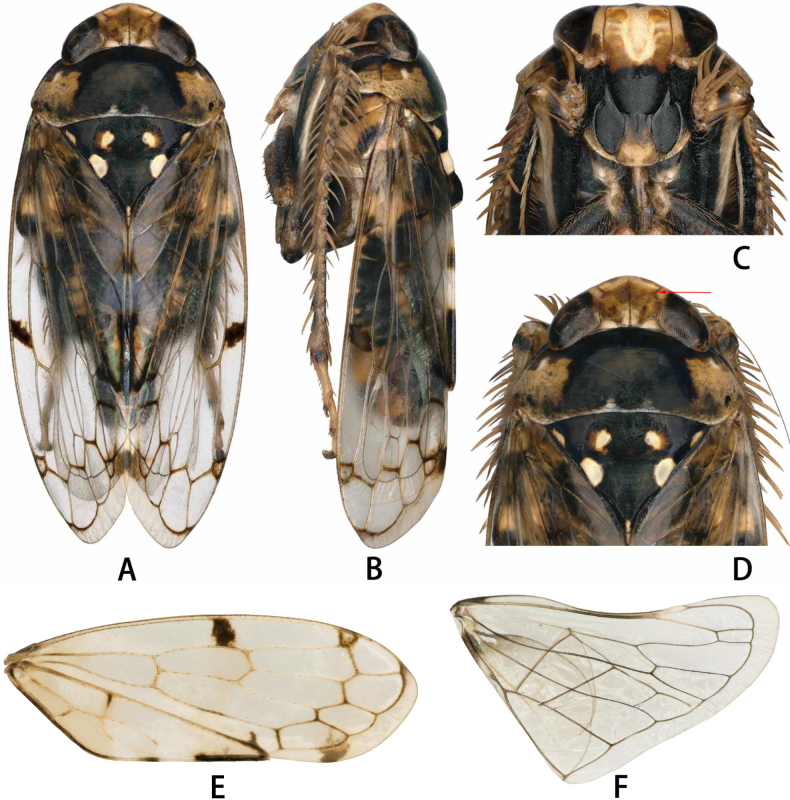
Male of *Minucella dilatata* sp. nov. **A**. Habitus, dorsal view; **B**. Habitus, lateral view; **C**. Face, ventral view; **D**. Head and thorax, dorsal view; **E**. Forewing; **F**. Hind wing.

This strikingly marked genus is similar to three other oriental stegelytrine genera, i.e. *Cyrta* Melichar, 1902, *Paracyrta* Wei, Webb & Zhang, 2008, and *Trunchinus* Zhang, Webb & Wei, 2007, in general appearance and in having the laterofrontal sutures extending well onto the vertex, with their apices converging (arrowed in Fig. 1D). *Minucella* differs from the above and other genera in the following characters: colour pattern consisting of dorsal symmetrical pale patches on the head and thorax (Fig. 1D) and face with gena and variably on other areas below antennae black (Fig. 1C); forewing with a very small 5th apical cell and claval veins fused (Fig. 1E); male pygofer with large, lobe-like, inner dorsal lateral process. Female first valvifer relatively long, visible in undissected specimens.

#### Description.

Colour pattern consisting of dorsal symmetrical, bright, pale patches on head and thorax (Fig. 1D), and face with gena and variably on other areas below antennae black (Fig. 1C); pronotum medially and scutellum black (Fig. 1D); forewings transparent, with a dark-brown, transverse band across corium from outer discal cell (Fig. 1A).

External features with head distinctly narrower than pronotum, with eyes laterally encroaching onto pronotum; vertex similar in length or slightly longer medially than next to eyes; coronal suture distinct, extending anteriorly to anterior margin of crown; antennae very long. Scutellum relatively long, medially keel-like (Fig. 1B). Forewing with inner margin of clavus elevated beyond scutellum (Fig. 1B); with a very small 5th apical cell and claval veins fused at midlength (Fig. 1E). Fore femur dorsally and anteriorly with numerous spine-like setae (Wei et al. 2008: figs 4, 5). Hind leg at rest with femur extending well forward to eye (Fig. 1D).

**Figure 2.**
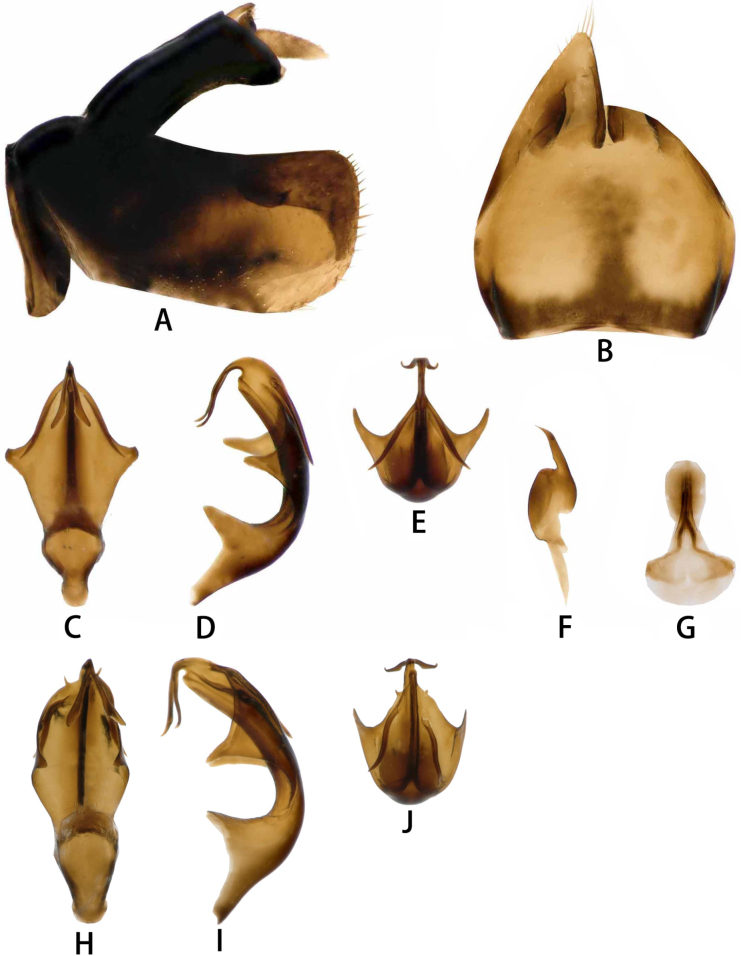
Male of *Minucella dilatata* sp. nov. **A**. Pygofer and anal tube, lateral view; **B**. Valve, ventral view; **C**. Subgenital plate, ventral view; **D**. Aedeagus, dorsal view; **E**. Aedeagus, lateral view; **F**. Aedeagus, posterior view; **G**. Style, dorsal view; **H**. Connective, dorsal view; **H–J**. Aedeagus, teratological form.

***Male genitalia***. Pygofer side usually rectangular, rarely triangular (in *M. angusta*), with a large, lobe-like inner dorsal process; dorsally and caudally with a few short, spine-like setae (Fig. 2A). Basal segment (Xth) of anal tube moderately long to long. Valve long and broad, laterally rounded (Fig. 2B). Subgenital plate short and triangular; apex with a few short, spine-like setae. Connective T-shaped, with stem longer than arms (Fig. 2G). Style with long outer basal arm and very short, lobe-like inner basal arm; apophysis moderately long and narrow, with apex turned laterally and finely pointed (Fig. 2F). Aedeagus with shaft antero-dorsally curved, cylindrical to laterally expanded, usually with two pairs of distal processes, rarely with three pairs of distal processes (in *M. elongata*), or with a single ventral basal process (in *M. divaricata*); basal apodeme moderately long. Dorsal connective between aedeagus and anal tube well developed (Wei et al. 2008: fig. 17).

***Female genitalia***. Female 7th sternite with posterior margin medially with a very distinct V-shaped incision and laterally with a rounded incision. First valvifer relatively long, visible in undissected specimen. Female pygofer with incurved ventroposterior margin. First valvulae sculpture comprising striations and scale-like rows, arranged longitudinally basally and oblique to transverse more distally. Second valvulae with blade-like area extending over distal half, relatively broad in lateral view, with teeth robust and truncate; dorsal sclerotised and hyaline areas absent. Third valvulae with ventral margin slightly incurved at midlength; expanded distal region extending over distal one-half.

#### Remarks.

Although some female specimens were collected during this study from Yunnan and Fujian Provinces, i.e. non-topotypical, they could not be associated with the males. This is because, although there are some differences in colour pattern between males of our new species, it has been shown that the colour pattern in species of this genus, i.e. in *M. leucomaculata*, is very variable (Wei et al. 2008: fig. 24), and therefore colour pattern alone is not a reliable feature to identify species. However, another possible character to identify females could be differences in the shape of the posterior margin of the seventh sternite. In this respect, our female specimens differ from the female of *M. leucomaculata* (Wei et al. 2008: fig. 19) in having the V-shaped medial incision and the curved lateral incision variably more pronounced. Therefore, if, in the future, females could be associated with males, either by finding male and female in copulation or by molecular studies, it might be possible to identify females based on the shape of the seventh sternite.

#### Distribution.

Oriental Region (China).

##### Key to species (♂) of *Minucella*

**Table:** 

1	Aedeagus shaft subbasally with a stout ventral process (Wei et al. 2008: fig. 34)	***M. divaricata***
–	Aedeagal shaft not as above	**2**
2	Aedeagal shaft with a lateral triangular expansion at midlength	**3**
–	Aedeagal shaft without a lateral triangular expansion at midlength	**4**
3	Aedeagal shaft with short apical processes arising from a narrow stalk (Fig. 2E) and longer subapical processes arising from ventral surface (Fig. 2C–E)	***M. dilatata* sp. nov**.
–	Aedeagal shaft with both short and long processes apically arising without stalk (Wei et al. 2008: figs 27, 28)	***M. leucomaculata***
4	Aedeagal shaft laterally expanded at apex with two pairs of processes (Fig. 3G–I)	***M. angusta* sp. nov**.
–	Aedeagal shaft not laterally expanded at apex with three pairs of processes (Fig. 4G–I)	***M. elongata* sp. nov**.

### Minucella dilatata sp. nov.

Taxon classificationAnimaliaHemipteraCicadellidae

9FA8D658-2082-54F7-A5BD-953E22C8D195

https://zoobank.org/52DB5918-7505-404D-BABA-9611BEA32010

Figs 1, 2

#### Description (male).

Colour and external features as in description. See also Fig. 1.

***Male genitalia***. Pygofer side approximately rectangular, basal and apical widths subequal; dorsal margin slightly concave medially (Fig. 2A). Basal segment (Xth) of anal tube very long. Aedeagus with shaft antero-dorsally curved, broadly laterally expanded, distally club-shaped in lateral view, thereafter abruptly narrowed with apical portion stalk-like and terminating in a pair of short slender processes; ventral surface of shaft with a pair of elongate, ventrally directed subapical processes (Fig. 2C–E). Valve broad; anterior margin medially slightly concave (Fig. 2B). Subgenital plate short; apical margin with several slender setae (Fig. 2B). Connective T-shaped; stem longer than arms (Fig. 2G). Style with elongated apical process, apically tapering (Fig. 2F).

#### Measurements (mm).

Length (including tegmen): ♂, 6.2–6.5.

#### Type material.

**China**: ***Holotype*** • ♂, Guizhou Prov., Shiqian County, Fodingshan, 16 August 2023, coll. Keting Duan (GUGC). ***Paratypes*** • ♂ Guizhou Prov., Fanjingshan, 30 July 2014, coll. Yunfei Wu (GUGC); • ♂ Guizhou Prov., Shiqian County, Fodingshan, 20 July 2025, coll. Huan Zhou (GUGC).

#### Distribution.

China: Guizhou.

#### Etymology.

The new species’ name is derived from the Latin word *dilatata*, meaning “expanded” or “widened”, referring to the distinctly laterally expanded margins of the aedeagus.

#### Remarks.

This species is similar to *M. leucomaculata* in the shape of the aedeagus but can be distinguished by having the distal processes mounted on a narrow stalk and the longer and more ventral processes arising from the ventral surface.

An additional specimen has been examined which is tentatively identified as this species with data: Guizhou Prov., Suiyang County, 16 July 2020, coll. Lan Zhang (GUGC). This specimen shows signs of deformity of the aedeagus resulting in asymmetry of the processes (Fig. 2H–J).

### Minucella angusta sp. nov.

Taxon classificationAnimaliaHemipteraCicadellidae

0E4FDBC1-D2FB-5B12-9C03-D97D768FD18C

https://zoobank.org/B07D45A6-1E72-4C90-A94D-00265ABEAB81

Fig. 3

#### Description (male).

**Figure 3.**
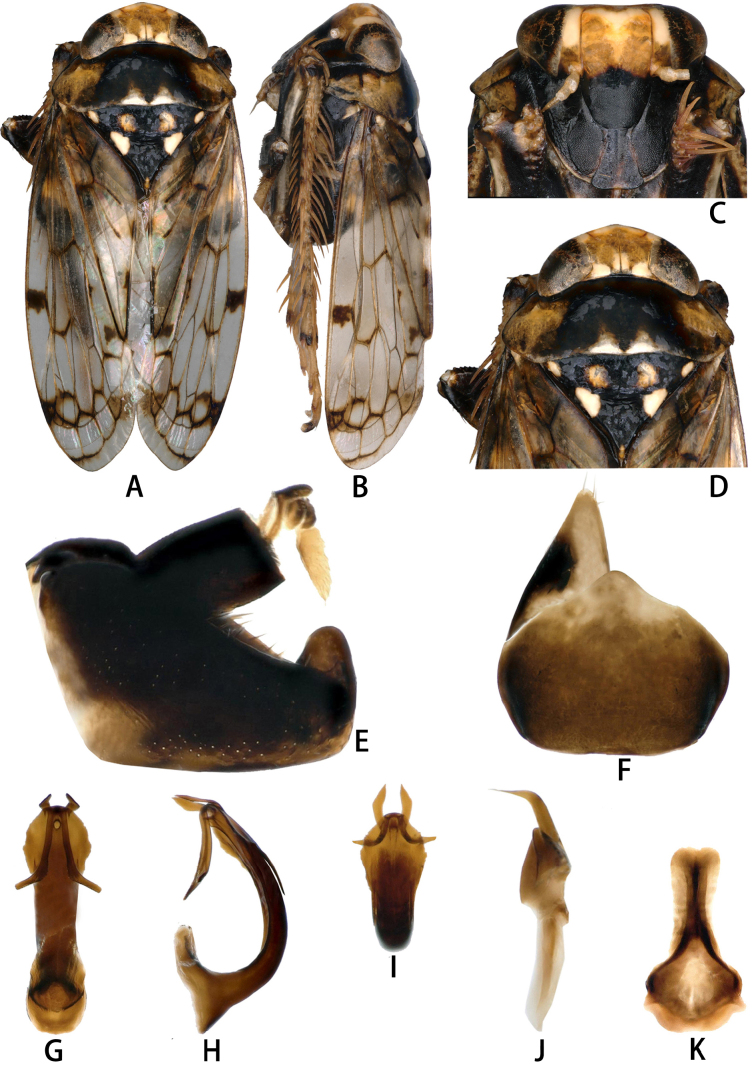
Male of *Minucella angusta* sp. nov. **A**. Habitus, dorsal view; **B**. Habitus, lateral view; **C**. Face, ventral view; **D**. Head and thorax, dorsal view; **E**. Pygofer and anal tube, lateral view; **F**. Valve and subgenital plate, ventral view; **G**. Aedeagus, dorsal view; **H**. Aedeagus, lateral view; **I**. Aedeagus, posterior view; **J**. Style, dorsal view; **K**. Connective, dorsal view.

Colour and external features as in description. See also Figure 3A–D.

***Male genitalia***. Pygofer side roughly triangular. Basal segment (Xth) of anal tube moderately long and robust (Fig. 3E). Aedeagus with shaft antero-dorsally curved, narrow in lateral view, and slightly laterally expanded, apically with two pairs of processes (Fig. 3G–I). Valve roughly triangular, its width greater than median length; posterior margin angularly medially produced (Fig. 3F). Subgenital plate short, basally broad, apically gradually narrowing, apical margin with several fine setae (Fig. 3F). Connective T-shaped (Fig. 3K). Style with apical process slender and pointed (Fig. 3J).

#### Measurements (mm).

Length (including tegmen): ♂, 5.4.

#### Type material.

**China**: ***Holotype*** • ♂, Fujian Prov., Wuyishan, 21 August 2013, coll. Yuan Liu (GUGC).

#### Distribution.

China: Fujian.

#### Etymology.

The new species name is derived from the Latin word *angusta*, meaning “narrow”, referring to the relatively narrow apical portion of the male pygofer side.

#### Remarks.

This species can be distinguished from other congeners in the male genitalia by the shape of the aedeagus (see Description and Key to species).

### Minucella elongata sp. nov.

Taxon classificationAnimaliaHemipteraCicadellidae

A8224EF2-AE68-5326-833D-88860EEDBE53

https://zoobank.org/9CA2D356-BB96-430A-9A1A-24576D8527C6

Fig. 4

#### Description (male).

**Figure 4.**
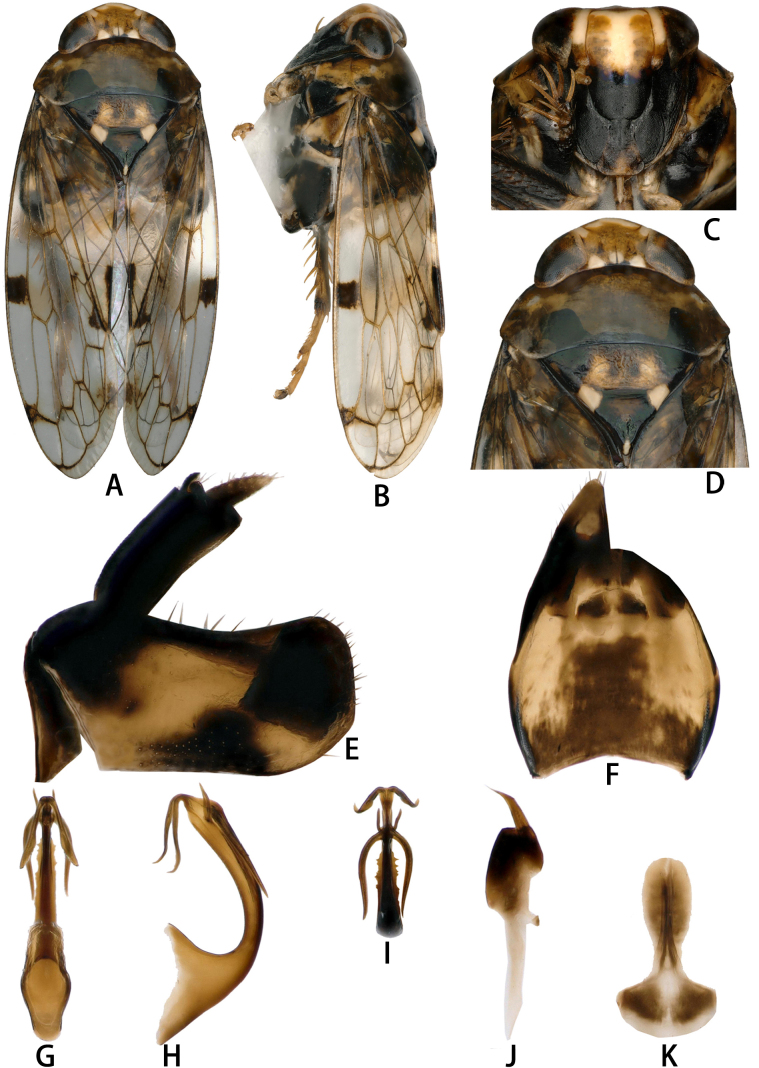
Male of *Minucella elongata* sp. nov. **A**. Habitus, dorsal view; **B**. Habitus, lateral view; **C**. Face, ventral view; **D**. Head and thorax, dorsal view; **E**. Pygofer and anal tube, lateral view; **F**. Valve and subgenital plate, ventral view; **G**. Aedeagus, dorsal view; **H**. Aedeagus, lateral view; **I**. Aedeagus, posterior view; **J**. Style, dorsal view; **K**. Connective, dorsal view.

Colour and external features as in Diagnosis. See also Figure 4A–D.

***Male genitalia***. Pygofer side approximately rectangular, basal and apical widths subequal; dorsal margin slightly medially concave (Fig. 4E). Basal segment (Xth) of anal tube very long. Aedeagus with shaft antero-dorsally curved, slightly laterally compressed, apex with a pair of processes on a narrow stalk; subapically with a pair of short, upward-directed, spine-like processes and a pair of long, ventrally directed processes (Fig. 4G-I). Valve elongate, its median length slightly greater than width (Fig. 4F). Connective T-shaped (Fig. 4K). Style apical process slender; preapical lobe well developed (Fig. 4J).

#### Measurements (mm).

Length (including tegmen): ♂, 6.0.

#### Type material.

**China**: ***Holotype*** • ♂, Guizhou Prov., Fanjingshan, 06 August 2023, coll. Shangmi Hu (GUGC).

#### Distribution.

China: Guizhou.

#### Etymology.

The new species name is derived from the Latin word *elongata*, meaning “long” or “elongated”, referring to the long, narrow aedeagal shaft in both lateral and ventral view.

#### Remarks.

This species can be distinguished from other congeners in the male genitalia by the shape of the aedeagus (see Description and Key to species).

## Supplementary Material

XML Treatment for Minucella

XML Treatment for Minucella dilatata

XML Treatment for Minucella angusta

XML Treatment for Minucella elongata

## Additional information

### Artificial Intelligence (AI) use

The authors accept full responsibility for the content of the manuscript, including the disclosure of any use of AI.

No AI tools were used in the preparation of this manuscript.

### Author ORCIDs

K-T. Duan https://orcid.org/0009-0008-8381-8890

Z.-Z. Li https://orcid.org/0000-0003-1609-9992

M.D. Webb https://orcid.org/0000-0002-1312-6142

J-C. Xing https://orcid.org/0000-0002-3738-7996

### Data availability

All of the data that support the findings of this study are available in the main text.

## References

[B1] Li ZZ, Dai RH, Xing JC (2011) Deltocephalinae from China (Hemiptera: Cicadellidae). Popular Science Press, Beijing, 336 pp. [In Chinese with English summary]

[B2] Li ZZ, Zhang B (2006) Description of three new species of the genus *Placidus* Distant from China (Hemiptera, Cicadellidae). Acta Zootaxonomica Sinica 31: 155–159. [In Chinese with English abstract]

[B3] Wei C, Zhang YL, Webb MD (2008) *Minucella*, a new leafhopper genus from China (Hemiptera: Cicadellidae: Stegelytrinae). Zootaxa 1854: 33–44. 10.11646/zootaxa.1854.1.3

